# Blood pressure and associated factors in a North African adolescent population. a national cross-sectional study in Tunisia

**DOI:** 10.1186/1471-2458-12-98

**Published:** 2012-02-03

**Authors:** Hajer Aounallah-Skhiri, Jalila El Ati, Pierre Traissac, Habiba Ben Romdhane, Sabrina Eymard-Duvernay, Francis Delpeuch, Noureddine Achour, Bernard Maire

**Affiliations:** 1 National Institute of Public Health (INSP), 5-7 rue El-Khartoum, Tunis, Tunisie; 2 Doctoral School 393, Université Pierre et Marie Curie, Paris, France; 3 National Institute of Nutrition and Food Technology (INNTA), 11 rue Jebel Lakhdar, Tunis, Tunisia; 4 IRD (Institut de Recherche pour le Developpement), UMR 204 NUTRIPASS, IRD-UM2-UM1, 911 av. Agropolis, 34394 Montpellier, France

**Keywords:** Adolescent, Blood pressure, Tunisia, Prevalence, Risk factors

## Abstract

**Background:**

In southern and eastern Mediterranean countries, changes in lifestyle and the increasing prevalence of excess weight in childhood are risk factors for high blood pressure (BP) during adolescence and adulthood. The aim of this study was to evaluate the BP status of Tunisian adolescents and to identify associated factors.

**Methods:**

A cross-sectional study in 2005, based on a national, stratified, random cluster sample of 1294 boys and 1576 girls aged 15-19 surveyed in home visits. The socio-economic and behavioral characteristics of the adolescents were recorded. Overweight/obesity were assessed by Body Mass Index (BMI) from measured height and weight (WHO, 2007), abdominal obesity by waist circumference (WC). BP was measured twice during the same visit. Elevated BP was systolic (SBP) or diastolic blood pressure (DBP) ≥ 90th of the international reference or ≥ 120/80 mm Hg for 15-17 y., and SBP/DBP ≥ 120/80 mm Hg for 18-19 y.; hypertension was SBP/DBP ≥ 95th for 15-17 y. and ≥ 140/90 mm Hg for 18-19 y. Adjusted associations were assessed by logistic regression.

**Results:**

The prevalence of elevated BP was 35.1%[32.9-37.4]: higher among boys (46.1% vs. 33.3%; *P *< 0.0001); 4.7%[3.8-5.9] of adolescents had hypertension. Associations adjusted for all covariates showed independent relationships with BMI and WC: - obesity vs. no excess weight increased elevated BP (boys OR = 2.1[1.0-4.2], girls OR = 2.3[1.3-3.9]) and hypertension (boys OR = 3.5[1.4-8.9], girls OR = 5.4[2.2-13.4]), - abdominal obesity (WC) was also associated with elevated BP in both genders (for boys: 2nd vs. 1st tertile OR = 1.7[1.3-2.3], 3rd vs.1st tertile OR = 2.8[1.9-4.2]; for girls: 2nd vs. 1st tertile OR = 1.6[1.2-2.1], 3rd vs.1st tertile OR = 2.1[1.5-3.0]) but only among boys for hypertension. Associations with other covariates were weaker: for boys, hypertension increased somewhat with sedentary lifestyle, while elevated BP was slightly more prevalent among urban girls and those not attending school.

**Conclusion:**

Within the limits of BP measurement on one visit only, these results suggest that Tunisian adolescents of both genders are likely not spared from early elevated BP. Though further assessment is likely needed, the strong association with overweight/obesity observed suggests that interventions aimed at changing lifestyles to reduce this main risk factor may also be appropriate for the prevention of elevated BP.

## Background

Hypertension is a major health problem throughout the world because of its high prevalence and its association with increased risk of cardiovascular diseases in the context of the epidemiological transition [1-4]. The presence of elevated blood pressure (BP) at a young age is a predictor of hypertension later in life [5] and it also has been reported that BP in children is associated with the same lifestyle factors as in adults, i.e. dietary habits [6-8], sedentary behaviors [9,10] and obesity [11-13]. Several studies have reported an increase in the prevalence of this disease in children and adolescents in many countries [14-17]. In Tunisia, where a nutritional transition is currently underway, 25.4% of 35-70 year-old adults were obese (37% for women vs. 13.3% for men), 61.6% were overweight (71.1% for women and 51.7% for men) [18] and 30.6% were found to be hypertensive in 2005 [19]; overweight was also prevalent among 15-19 year-old adolescents and affected 19% of them (17.4% for boys and 20.7% for girls) [20]. However, up to now, no study has attempted to assess the extent of elevated BP at a large scale among Tunisian adolescents. In the context of increasing prevalence of obesity in Tunisia, the aim of this study was to assess BP status in male and female Tunisian adolescents, aged 15-19 years, and to investigate environmental, anthropometric, socio-economic and behavioral (physical activity, perceived stress) associated factors.

## Methods

### Study area

Tunisia is a North African country of about ten million inhabitants [21], that has undergone a steady and rapid economic development and reached an upper middle level of development (ranked 89th out of 177 countries on the Human Development Index composite scale in 2005) [22], but which 7 administrative regions are geographically and socio-economically contrasted.

### Study design and subjects

The national cross-sectional survey of adolescents aged 15 to 19 years was carried out from April to September, 2005. Based on the most recent population census in 2004, the two-stage random clustered sample of households was stratified according to the seven administrative regions of Tunisia. Next, 47 census districts were randomly selected in each region, with a probability proportional to size in number of households. At the second stage, 20 households were sampled randomly in each selected district and all 15-19-year olds living in these households (6580) at the time of the survey were eligible [20]. With reference to the 0.48 ratio of 15-19-year olds per household (2004 Tunisian census), about 3138 subjects were thus expected to be included.

### Data collection and measurements

Data on demographic characteristics, area (rural vs. urban), household economic status, whether the adolescent was registered at school or not at the time of the survey, low physical activity, and perceived stress were collected using interviewer-administrated questionnaires.

Trained investigators took anthropometric and blood pressure (BP) measurements using standardized procedures.

A household economic score was computed by correspondence analysis from the matrix of binary variables coding for type of house, number of people per room, type of sanitation, type of drinking water supply and possessions such as car, television, computer, satellite dish and refrigerator. This score is the coordinate on the first axis of the correspondence analysis and used as a proxy indicator of the economic level of the household. We identified three levels by dividing the score into tertiles of increasing economic level [23,24].

Adolescents were categorized according to whether they were attending school or not at the time of the survey.

Physical activity practised during the month preceding the survey was estimated using a validated frequency questionnaire [20,25]. The international compendium of physical activities was used for the calculation of the metabolic equivalent (MET) of each daily activity [26]. The intensity of the physical activities was classified as light, moderate or vigorous (respectively < 3, 3-6 and > 6 Mets; 1 Met = 3.5 mL O^2^/kg) using the CDC-American College of Sports Medicine classification [27]. The percentage of low intensity daytime physical activities was computed and coded into tertiles, with sedentary lifestyle increasing from the 1st to the 3rd tertile.

The perceived stress score was calculated using a validated stress scale composed of four questions [28]. Each question had five answers coded from 0 to 100. The perceived stress score is the arithmetic mean of the four sub-scores; it increases with the level of perceived stress. For analysis purposes the score was divided in three tertiles of increasing stress level.

#### Anthropometry

Weight was measured to the nearest 0.1 kg using electronic scales (Teraillon, France) with regular checks for accuracy and precision. Height was measured to the nearest millimetre using portable gauges (Seca, Germany), with the subject in a standing position, without shoes. Abdominal fat was assessed by waist circumference (WC) that was measured to the nearest 0.1 cm using a non-elastic metric measuring tape. Assessment of overweight status was based on the age and sex-specific body mass index (BMI, kg/m^2^) reference distributions developed by the World Health Organisation [29]. The cut-offs were BMI ≤ +1 standard deviation (SD) for "no excess weight"; "+1 SD < BMI ≤ +2 SD" for overweight (but not obese) and BMI > +2 SD for obesity. WC was coded in tertiles (separately for each gender).

#### Blood pressure

BP was measured twice by the auscultatory method using a stethoscope and calibrated sphygmomanometers (Vaquez Laubry type, Spengler, France): the first measurement after 10 min of rest and the second at the end of the interview (an average of 30 min later). The estimation of BP was based on the average of the two measurements. For 15-17 year olds, pre-hypertension (pre-HT) was defined as average systolic BP (SBP) and/or diastolic BP (DBP) ≥ 90th age, sex and height specific percentile (or ≥ 120/80 mm Hg) but < 95th percentile; hypertension (HT) as average SBP and/or DBP levels ≥ 95th percentile of the reference values for age, sex and height according to the standard definition introduced in 2003 and extended to children and adolescents in 2004 [30]. For 18-19 year olds, pre-HT was defined as average SBP/DBP ≥ 120/80 mm Hg but < 140/90 mm Hg; HT as SBP/DBP ≥ 140/90 mm Hg [31,32]. Hence, we defined elevated BP as SBP/DBP ≥ 90th percentile (or above 120/80 mm Hg).

### Ethics

The protocol of the survey was reviewed and approved by the Ethics Committee on Human Research of the National Institute of Nutrition and the Tunisian National Council of Statistics (visa n°5/2005). Subjects were informed of their right to refuse and of the strict respect of the confidentiality of their answers. All adolescents and their parents gave their verbal consent.

### Data management and statistical analysis

Epidata software, version 3.1 was used for data entry and validation [33] and Stata 11 for data management and analysis [34]. The type I error risk was set at 0.05. All analyses (descriptive, crude and adjusted associations) took the sampling design (stratification, clustering and sampling weights) into account.

Due to gender differences underlined in preliminary analyses, all analyses were performed separately for each gender. Descriptive analysis of the sample including demographic, socio- economic, anthropometric and BP characteristics was performed using t-tests to compare means and chi-square tests to compare percentages between genders. We studied unadjusted associations between socio-demographic, lifestyle factors and anthropometric status and blood pressure status (prevalence of elevated BP and HT) using chi-square tests. Local polynomial smoothing was used to graphically assess the unadjusted relationship between BMI and WC and elevated BP and hypertension by gender.

Multivariate logistic regression models were fitted to assess adjusted associations between covariates and elevated BP or hypertension: covariates were age (interval), area (urban vs. rural), household economic level (in tertiles), school status (attending school vs. not), percentage of low intensity daily physical activity (in tertiles), perceived stress score (in tertiles), BMI (no excess weight/overweight/obesity), WC (tertiles by gender). We first fitted separate models to assess association of BMI (respectively WC) with BP separately (adjusting for age and all socioeconomic and behavioral characteristics). In the second step, we performed the analyses using both WC and BMI simultaneously in the models.

## Results

### Sample characteristics

Taking into account refusals, absences and missing BP data, 2870 out of the expected 3138 15-19-year old subjects were used in the analyses i.e. an overall response rate of 91.5%.

Socio-demographic characteristics, lifestyle factors, anthropometric and BP status for boys and girls are shown in Table 1. The weighted estimate of the proportion of females was 49% but there was nonetheless a substantially lower response rate for males. About two thirds of the adolescents lived in urban areas. One third of the adolescents were not attending school during the study period with a higher percentage of boys. The household economic level was similar among boys and girls. Compared to boys, girls had a more sedentary lifestyle and higher level of perceived stress. Mean BMI (kg/m^2^) was higher for females (22.0 ± 0.1) than for males (20.9 ± 0.1). The prevalence of overweight (not including obesity) among adolescents was 20.1% ± 1.0 and obesity prevalence was 5.0% ± 0.6 with no statistically significant difference between boys and girls. Mean waist circumference (cm) was 75.7 ± 0.4 for boys and 73.1 ± 0.3 for girls. The mean systolic and diastolic BP (mmHg) was higher in males: the mean SBP was 114.2 ± 0.4 for boys and 110.9 ± 0.4 for girls (*P *< 0.0001) and the mean DBP was 68.1 ± 0.3 for boys and 66.6 ± 0.4 for girls (*P *< 0.001). The prevalence of elevated BP was 39.8% [37.4-42.3], higher among boys (46.1% [42.7-49.5]) than among girls (33.3% [30.0-36.7]; *P *< 0.0001). One third of the adolescents had a pre-HT (35.1% [32.9-37.4]), and pre-HT was more frequent among boys (41.8% [38.5-45.1]) than girls (28.2% [25.2-31.4]; *P *< 0.0001). HT was present in 4.7% [3.8-5.9] of adolescents with no significant difference between boys and girls.

**Table 1:** Characteristics of the sample of 15-19 y, Tunisian adolescents (n = 2870).

		**Boys**		**Girls**	
		
**Characteristics**	**n**^**1**^	**Mean(s.e.)** **or** **%**^**2**^	**n**^**1**^	**Mean(s.e.)** **or** **%**^**2**^	***P*-value**^**3**^
		
	1294	51.0	1576	49.0	
**Age (years)**					
*15-17*	838	63.2	982	63.2	0.99
*18-19*	456	36.8	594	36.8	
**Area**					
*Urban*	707	62.3	825	62.4	0.93
*Rural*	587	37.7	751	37.6	
**Socio-economic factors**					
Household's economic level					
*Low*	526	38.4	645	37.4	
*Intermediate*	427	32.1	552	36.0	0.14
*High*	283	29.5	299	26.6	
Attending school	1292	69.1	1572	74.2	0.020
**Behavioral factors**					
Proportion of low physical activity					
*1^st ^tertile (low)*	562	46.2	283	20.0	
*2^nd ^tertile (middle)*	413	31.0	543	35.8	< 10^-4^
*3^rd ^tertile (high)*	319	22.8	750	44.3	
Perceived stress					
*1^st ^tertile (low)*	473	37.7	504	31.6	
*2^nd ^tertile (middle)*	474	35.9	566	36.0	0.0021
*3^rd ^tertile (high)*	341	26.4	500	32.4	
**Anthropometric characteristics**					
Body Mass Index (kg/m^2^)	1294	20.9 (0.1)	1576	22.0 (0.1)	< 10^-4^
*No excess weight*	1011	76.9	1161	72.9	
*Overweight*	218	17.9	337	22.3	0.085
*Obesity*	65	5.2	78	4.8	
Waist circumference (cm)	1287	75.7 (0.4)	1571	73.1 (0.3)	< 10^-4^
*1^st ^tertile*	496	33.4	632	36.7	
*2^nd ^tertile*	433	35.3	468	31.7	0.14
*3^rd ^tertile*	358	31.3	471	31.7	
**Blood pressure (BP) status**					
Systolic BP (mmHg)	1294	114.2 (0.4)	1576	110.9 (0.4)	< 10^-4^
Diastolic BP (mmHg)	1294	68.1 (0.3)	1576	66.6 (0.4)	< 10^-3^
Elevated BP	1294	46.1	1576	33.3	< 10^-4^
Hypertension	1294	4.3	1576	5.1	0.45

1-Number of subjects surveyed

2-Weighted mean (standard error) or proportion (accounting for unequal probabilities of selection and/or differential response rates)

3- Boys vs. girls (chi-square test for %, *t*-test for means)

### Univariate analysis

**Figure 1:**
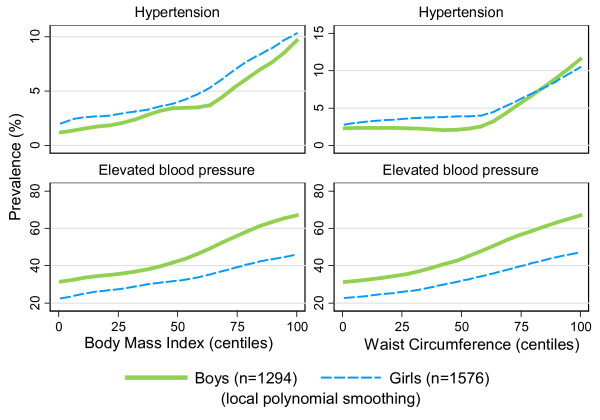
**Prevalence of elevated blood pressure and hypertension according to body mass index and waist circumference**. By gender, prevalence of elevated blood pressure and hypertension according to body mass index and waist circumference (in centiles) among Tunisian 15-19 y. adolescents. Overall trends are estimated using local polynomial smoothing.

For both genders, the prevalence of elevated BP increased quite linearly with BMI (*P *< 0.0001), the association being somewhat steeper for boys (Figure 1); a significant association was also found with WC for both genders (*P *< 0.0001). Association of BMI and WC with hypertension was also marked for both genders, these associations being stronger after the 50th centile of BMI and especially for WC (Figure 1). In accordance, prevalence of elevated BP and HT increased markedly with BMI categories; a similar association was found for elevated BP with WC tertiles but the prevalence of HT only increased in the third tercile of WC (vs. 1st and 2nd tertiles, Table 2). Relationship between BMI and WC was obviously strong for both genders as the linear correlation coefficient was 0.72 (*P *< 0.0001) for boys and 0.66 (*P *< 0.0001) for girls.

**Table 2:** Unadjusted associations of socio-demographic and lifestyle factors, Body Mass Index and Waist Circumference with blood pressure

	Boys						Girls			
	**Elevated BP**^**1**^			Hypertension			**Elevated BP**^**1**^		Hypertension	
	n	**%**^**2**^	**P**^**3**^	**%**^**2**^	**P**^**3**^	n	**%**^**2**^	**P**^**3**^	**%**^**2**^	**P**^**3**^
**Area**										
*Urban*	707	46.6	0.69	4.4	0.90	825	29.0	0.0010	3.8	0.011
*Rural*	587	45.2		4.2		751	40.4		7.3	
**Household's economic level**										
*Low*	526	45.6		3.0		645	37.9		6.5	
*Intermediate*	427	44.0	0.44	4.2	0.35	552	33.2	0.037	4.5	0.068
*High*	283	49.5		5.5		299	27.4		2.6	
**Attending school**										
*Yes*	893	44.1	0.079	4.2	0.73	1151	30.6	0.0012	4.1	0.003
*No*	399	50.8		4.7		421	41.2		8.3	
**Proportion of low physical activity**										
*1^st ^tertile (low)*	562	44.5		2.3		283	30.3		5.6	
*2^nd ^tertile (middle)*	413	46.3	0.56	5.0	0.0096	543	31.6	0.18	3.6	0.13
*3^rd ^tertile (high)*	319	49.0		7.4		750	36.0		6.1	
**Perceived stress**										
*1^st ^tertile (low)*	473	44.6		4.0		504	33.3		5.8	
*2^nd ^tertile (middle)*	474	47.0	0.82	5.6	0.30	566	35.8	0.33	3.8	0.25
*3^rd ^tertile (high)*	341	46.1		3.0		500	30.6		5.9	
**Body Mass Index**										
*No excess weight*	1011	39.6		2.7		1161	28.9		3.3	
*Overweight*	218	65.9	< 10^-4^	4.8	< 10^-4^	337	43.5	< 10^-4^	8.7	< 10^-4^
*Obesity*	65	74.4		26.0		78	52.8		15.3	
**Waist Circumference**										
*1^st ^tertile*	496	31.6		2.5		632	23.8		3.5	
*2^nd ^tertile*	433	43.9	< 10^-4^	1.4	< 10^-4^	468	32.6	< 10^-4^	3.5	< 10^-3^
*3^rd ^tertile*	358	64.1		9.7		471	44.8		8.7	

1-Blood Pressure

2-Weighted prevalences (accounting for unequal probabilities of selection and/or differential response rates)

3-*P*-value for unadjusted association between elevated BP or hypertension and variable in left column

Regarding crude associations with socio-demographic and lifestyle factors, for boys, the prevalence of HT increased with tertiles of low daily physical activity (Table 2). For girls, the prevalence of elevated BP and HT was higher in rural than urban settings, and among those who were not attending school. Prevalence of elevated BP decreased somewhat with higher household economic levels (Table 2).

### Multivariate analysis

In analyses (detailed data not shown), adjusted for all socio-economic and behavioral covariates but including only one anthropometric index at a time, obesity vs. no excess weight markedly increased the prevalence of HT (boys OR = 8.4[3.7-18.8], girls OR = 7.2[3.2-16.1]; *P *< 10^-4^) and that of elevated BP (boys OR = 3.7[2.0-6.9], girls OR = 3.4[2.1-5.6]; *P *< 10^-4^); similar though weaker associations were observed with overweight. WC was also associated with elevated BP in both genders (for boys: 2nd tertile vs. 1st tertile OR = 1.7[1.3-2.4], 3rd tertile vs.1st tertile OR = 4.0[2.9-5.5] *P *< 10^-4^; for girls: 2nd tertile vs. 1st tertile OR = 1.7[1.2-2.2], 3rd tertile vs.1st tertile OR = 2.8[2.0-3.8] *P *< 10^-4^); a similar but weaker association was observed with HT for boys and girls.

In adjusted analyses including simultaneously BMI and WC in the models (Table 3), the strength of the association with BMI and WC decreased somewhat: but being obese vs. having no excess weight still markedly increased HT (boys OR = 3.5[1.4-8.9], girls OR = 5.4[2.2-13.4]) as well as elevated BP (boys OR = 2.1[1.0-4.2], girls OR = 2.3[1.3-3.9]); analogous though weaker associations were observed with overweight. WC was also still associated with elevated BP in both sexes (for boys: 2nd tertile vs. 1st tertile OR = 1.7[1.3-2.3], 3rd tertile vs.1st tertile OR = 2.8[1.9-4.2]; for girls: 2nd tertile vs. 1st tertile OR = 1.6[1.2-2.1], 3rd tertile vs.1st tertile OR = 2.1[1.5-3.0]); a similar but weaker association was observed with HT in boys only. Adjusted associations with socio-economic and behavioral covariates were less straightforward and varied by gender: for boys, HT somewhat increased with sedentary lifetsyle (medium tertile vs. low OR = 2.3[1.0-4.9], high vs. low OR = 2.8[1.1-7.0]), for girls elevated BP was slightly more prevalent in urban settings (urban vs. rural OR = 1.5[1.1-2.1]) and among those who were not attending school (OR = 1.4[1.0-1.8]) or had a more sedentary lifestyle (high vs. low OR = 1.4[1.0-1.9]).

**Table 3:** Adjusted associations of socio-demographic and lifestyle factors, Body Mass Index and Waist Circumference with blood pressure

	Boys(n = 1221^1^)				Girls(n = 1484^1^)			
	**Elevated BP**^**2**^		Hypertension		**Elevated BP**^**2**^		Hypertension	
	**Adjusted OR**^**3**^ (CI95%)	**P**^**4**^	**Adjusted OR**^**3**^ (CI95%)	**P**^**4**^	**Adjusted OR**^**3**^ (CI95%)	**P**^**4**^	**Adjusted OR**^**3**^ (CI95%)	**P**^**4**^
**Area**								
*Urban*	1	0.49	1	0.22	1	0.021	1	0.081
*Rural*	1.1 (0.8-1.5)		1.6 (0.8-3.5)		1.5 (1.1-2.1)		1.6 (0.9-2.8)	
**Household's economic level**								
*Low*	1		1		1		1	
*Intermediate*	0.8 (0.6-1.1)	0.29	1.6 (0.7-3.7)	0.27	0.9 (0.7-1.3)	0.52	0.8 (0.5-1.5)	0.62
*High*	1.0 (0.7-1.5)		2.1 (0.8-5.5)		0.8 (0.5-1.2)		0.6 (0.2-1.8)	
**Attending school**								
*Yes*	1	0.065	1	0.32	1	0.033	1	0.088
*No*	1.4 (1.0-2.0)		1.5 (0.7-3.4)		1.4 (1.0-1.8)		1.8 (0.9-3.5)	
**Proportion of low physical activity**								
*1^st ^tertile (low)*	1		1		1		1	
*2^nd ^tertile (middle)*	1.2 (0.9-1.6)	0.35	2.3 (1.0-4.9)	0.072	1.1 (0.8-1.6)	0.054	0.7 (0.3-1.4)	0.075
*3^rd ^tertile (high)*	1.3 (0.9-1.8)		2.8 (1.1-7.0)		1.4 (1.0-1.9)		1.4 (0.7-2.9)	
**Perceived stress**								
*1^st ^tertile (low)*	1		1		1		1	
*2^nd ^tertile (middle)*	1.0 (0.7-1.4)	0.88	1.1 (0.5-2.2)	0.45	1.1 (0.8-1.6)	0.54	0.7 (0.3-1.3)	0.25
*3^rd ^tertile (high)*	0.9 (0.7-1.3)		0.7 (0.3-1.6)		1.0 (0.7-1.4)		1.1 (0.6-2.0)	
**Body Mass Index**								
*No excess weight*	1		1		1		1	
*Overweight*	2.0 (1.3-2.9)	0.0014	0.9 (0.4-2.4)	0.012	1.5 (1.1-2.1)	0.0028	2.1 (1.0-4.3)	0.0017
*Obesity*	2.1 (1.0-4.2)		3.5 (1.4-8.9)		2.3 (1.3-3.9)		5.4 (2.2-13.4)	
**Waist circumference**								
*1^st ^tertile*	1		1		1		1	
*2^nd ^tertile*	1.7 (1.3-2.3)	< 10^-4^	0.5 (0.2-1.7)	0.0046	1.6 (1.2-2.1)	0.0001	1.0 (0.4, 2.2)	0.50
*3^rd ^tertile*	2.8 (1.9-4.2)		2.7 (1.1-6.6)		2.1 (1.5-3.0)		1.5 (0.7-3.2)	

1-Complete case analysis

2- Blood pressure

3- OR: Odds-Ratio, adjusted for age and all the variables in the first column

4- Adjusted for age and all the variables in the first column

## Discussion

The main results of this national study showed that elevated BP was prevalent among Tunisian adolescents, more so in boys, that prevalence increased strongly with BMI and WC, while associations with behavioral and socio-economic factors were much less straightforward.

Apart from a possible overestimation of BP due to methodology limits, the observed prevalence of pre-HT and HT in Tunisian adolescents are among the highest rates reported in the literature for this age class, where prevalence ranged between < 1% and 5.1% for HT [15,32,35] and from < 10% to < 40% for pre-HT [32,36-39]. Comparing the prevalence of elevated BP with data in the literature is difficult because it is easily subject to bias related to the class of age chosen as well as regional differences in the definition of elevated BP, the distribution of reference BP data and the method used to measure BP [31,32,40]. However in many countries, it remains largely underestimated [41,42]. Recently, Hansen et al. [42] showed that among a cohort of 14187 children and adolescents aged 3-18 followed regularly in a large academic urban medical system in northeast Ohio (USA), only 26% of HT and 11% of pre-HT cases had an appropriate diagnosis recorded in their electronic medical record. The authors suggested that this low diagnosis rate could be accounted for by the lack of knowledge concerning normal BP ranges and the lack of awareness of a patient's previous BP readings. Thus, it would be useful to provide a practical tool for physicians to enable them to easily identify the threshold of normal BP values for adolescents according to age, gender and height.

The present study identified gender differences mainly in the prevalence of elevated BP as Tunisian males were more affected than females. Gender differences in the risk of developing elevated BP have been reported by several authors in different populations [37,43-45]. According to Dasgupta et al., the gender differences in the risk of elevated BP can be explained by the impact of sex steroids on BP [44]. This factor is also strongly suggested by experimental models [46].

The prevalence of elevated BP and HT were higher among overweight and obese Tunisian adolescents. The association between BP level and overweight status has been documented in many young populations thanks to prospective and cross-sectional studies [11,32,47-49]. According to a recent review, there are three main mechanisms of obesity-induced- hypertension: activation of the sympathetic nervous system, renal, and hormonal dysfunction [50].

On the other hand, in the models including both BMI and WC as covariates, a significant association was found between WC and BP status for males and females adolescents, especially with elevated BP. These results show that BMI and WC, despite being strongly correlated, once adjusted for one another, remain significantly associated with elevated BP, indicating that they are independently associated with elevated BP. These findings are in agreement with data in the literature [49,51-53]. Indeed, according to many authors, the distribution of body fat in particular abdominal fat, is even considered to be more predictive of health risk than whole body composition measures such as BMI [54]. The association between WC and elevated BP has been attributed to hyperinsulinemia induced by excess abdominal fat [55,56].

Comparison (detailed data not shown) of our BP data with data gathered among Tunisian adolescents (aged 15-19) during the Tunisian 1997 national survey using comparable data (1996 BP data featuring only one measurement compared to the first measurement of our 2005 survey), showed that SBP and DBP had decreased in girls but remained stable in boys. Adjustment for BMI, age, height, and area (urban/rural) did not change these trends. BMI is a major determinant of BP, but the absence of an increase in BP over the time in Tunisia and reported in several other studies, suggests that, despite the significant increases in the prevalence of overweight [57], other factors may also have an influence on the evolution of BP [58]. Such factors may include diet characteristics, i.e. the intake of fruits, vegetables, or dairy products, which leads to an increase in calcium consumption, which traditionally was rather low; as a matter of fact, increased consumption of dairy products has been repeatedly associated with reduced risk of elevated BP [59-61]. In accordance, the assessment of dietary intake in a subsample of the same subjects [62], underlined a main dietary pattern of modernization associated with urbanization and regional socio-economic development, this pattern being associated with a favorable effect on BP in girls.

According to some authors, studies using uniformly standardized methodology showed a positive trend in the prevalence of HT among adolescents especially with increasing prevalence of obesity, in contrast to other studies which showed a decrease [32]. On the other hand, Chiolero et al. showed that at the population level, a marked increase in the prevalence of obesity in children and adolescents in a rapidly developing country was not associated with a commensurate secular rise in mean BP [58-61,63] despite the strong relationship between obesity and elevated BP at individual level in the same study. On the other hand, some authors showed that obesity during childhood is associated with elevated BP in early adulthood [64,65]. All these results lead us to formulate the following hypothesis: persistent exposure to overweight is a risk factor for elevated BP later; the association observed at individual level may be explained by premature exposure to overweight in early childhood and the recent exposure to overweight could expose the subjects to the risk of elevated BP in early adulthood. Hence, the effect of the increase in the prevalence of overweight in Tunisian adolescents will probably lead to an increase in the prevalence of BP later in adulthood.

In our study, elevated BP and/or HT among males and females was not strongly associated with the other factors we analyzed (socio-economic factors, physical activity, stress level) as much as with intermediate outcomes such as BMI and WC. Prevalence of elevated BP increased somewhat with an increase in sedentarity level among Tunisian adolescents; this result is consistent with most other studies [37,44,63,66,67]. Hence, the promotion of weight control using appropriate strategies (measures that target environmental factors as well as behavioral ones) in Tunisian children and adolescents aimed at reducing overweight, could also help reduce elevated BP and many other risk factors of chronic diseases during adolescence and adulthood. Our data did not reveal any link between perceived stress and BP. Indeed, there is a controversy in the literature about this relationship as some studies based on declared perceived stress report decreasing associations [68-70].

The absence of monitoring of BP among adolescents reflects the facts that the majority of physicians and parents in Tunisia think that elevated BP is rare among adolescents and are probably not aware of current epidemiologic trends in adolescents' health. Indeed, the prevalence of overweight and obesity (risk factors for elevated BP) among adolescents has increased in Tunisia [20], due to the epidemiological transition and its impact on the environment and changes in lifestyle: indeed the analysis of changes in the food consumption behavior among 15-19-year old Tunisian adolescents between 1997 and 2005 showed for instance that the intake of total fat, saturated fat and total sugars increased while the intake of PUFAs decreased [57]. Pre-HT left untreated during adolescence predisposes to persistent HT in adulthood [71]. Thus, it is reasonable to take action to prevent elevated BP during childhood and adolescence and to contribute to reducing morbidity and mortality related mainly to cardiovascular diseases. However, whereas screening should be focused on overweight and obese young people; primary prevention should concern the whole young population.

As for its strengths and limitations, the study was based on a large random sample of the Tunisian adolescents, the first at such a large scale in Tunisia. However, the cross-sectional design has known limitations regarding causal interpretations of observed associations between the measured covariates and elevated BP and HT. For financial and practical reasons, assessment of the BP status, was based on two measurements of BP made during the same visit. Indeed, we measured BP in non-stress conditions (at home, no white coat), and a recent study showed that BP screening based on three or more measurements per visit was no better than two [72], but not using sets of measurements made during repeated visits as advised in the literature [30], may have led to overestimation of elevated BP [15]. Not taking into account dietary habits was another obvious limitation of this study but this aspect had already partly been dealt with by the same authors though only on a sub-sample of the subjects [62].

## Conclusion

Within the limits of the results based on BP measurement on one visit only, our results suggest that Tunisian adolescents of both genders are likely not spared from early elevated BP. Though no clear-cut associations between BP and environmental or behavioral factors were identified in the study and further studies are needed, the strong association with overweight or obesity suggest that interventions aimed at lifestyle modifications to reduce that main risk factor could be also useful in the prevention of HT or elevated BP among Tunisian adolescents and thus reducing the risk of associated diseases when this generation reaches adulthood.

## Competing interests

The authors declare that they have no competing interests.

## Authors' contributions

HAS designed the study, supervised data collection, planned and performed data analysis and drafted the manuscript; JEA and PT helped interpret the results and write the manuscript; SED helped with data management; HBR and NA contributed to the study design, FD contributed to the study design and to writing the manuscript; and BM was involved in all steps, from the design of the study to the revision of the manuscript. All authors read and approved the manuscript.

## Pre-publication history

The pre-publication history for this paper can be accessed here:

http://www.biomedcentral.com/1471-2458/12/98/prepub
